# Case Report: Tropical sprue, diagnostic challenges of an old but unrecognized disease

**DOI:** 10.12688/f1000research.125305.2

**Published:** 2023-09-13

**Authors:** Jihene Guissouma, Hana Ben Ali, Hend Allouche, Insaf Trabelsi, Olfa Hammami, Yosra Yahia, Ilhem Mchirgui, Marwa Mabrouk, Hatem Ghadhoune

**Affiliations:** 1 Faculty of medicine of Tunis, Tunis El Manar University, Tunis, 1007, Tunisia; 2 Medical intensive care unit, Habib Bougatfa university hospital, Bizerte, 7021, Tunisia; 3 Pediatric department, Habib Bougatfa university hospital, Bizerte, 7021, Tunisia; 4 Emergency depatement, Rabta University Hospital, Tunis, 1007, Tunisia; 5 Emergency department, Habib Bougatfa university Hospital, Bizerte, 7021, Tunisia

**Keywords:** Chronic diarrhea, small bowel disease, malabsorption, tropical sprue, villous atrophy.

## Abstract

Tropical sprue (TS) is a post-infective disease of the small bowel characterized by a malabsorption syndrome affecting tropics inhabitants and visitors. Diagnosis of TS remains challenging since it can be confused with common diarrheal diseases, especially in non-endemic areas. We report a Tunisian case of latent TS.

A 58-year-old male with a history of chronic watery diarrhea, was admitted to the intensive care unit for confusion which was related to a severe metabolic acidosis. Despite the neurological improvement after hydro-electrolytic resuscitation and acid-base disorders correction, the patient continued to have three to five loose stools daily.

A nutritional assessment showed a malabsorption syndrome: iron, Vitamin B12and folate deficiencies; normochromic normocytic anemia and hypoalbuminemia.

Gastrointestinal endoscopy showed duodenal villous atrophy and biopsy confirmed subtotal villous atrophy with increased intraepithelial lymphocytosis and a thickened hyalonalized sub-epithelial basal lamina. Celiac disease was evoked, however the patient did not improve on a gluten-free diet and the celiac serology was negative.

On re-interviewing, we discovered that the patient had spent two months in India three years prior.

Given the travel history, clinico-biological and histological data TS was highly considered and a good response to a five-month antibiotic course combined to nutritional supplementation supported this diagnosis.

Clinico-biological, endoscopic and histological findings were overlapping between TS and other malabsorption diseases, explaining diagnosis difficulties. TS should be systematically discussed in tropics visitors presenting with chronic diarrhea. Improvement after micronutrient and vitamin deficiencies replacement combined to a prolonged antibiotic course supports the diagnosis of TS.

## Introduction

Tropical sprue (TS) is a very old disease. The first reported cases were described by a Yorkshireman, Dr William Hillary, among the inhabitants of the island of Barbados over 200 years ago. ^1^ Endemic TS occurs in the tropical and subtropical regions but is underrecognized elsewhere. It is predominantly present in southern and southeast Asia and the Caribbean Islands, and rarely described in North Africa and Europe. ^2^ Within the present era of globalization and worldwide travel, it is important for all clinicians to be aware of the possibility of TS in patients presenting with diarrhea, malabsorption, multiple nutritional deficiencies and mucosal abnormalities in the small bowel who have travelled to endemic regions. ^3^

Diagnosis of TS remains challenging since it can be confused with common diarrheal diseases, including celiac disease, Crohn’s disease, bacterial overgrowth, and other infectious aetiologies. ^4^

In this article, we present a Tunisian case of latent TS revealed by a confusional syndrome.

## Case report

A 58-year-old North African male was admitted to the intensive care unit for confusion without fever. He was single, working as a trader and had a history of iron deficiency anemia. There were no particular family antecedents. The acute symptoms had been preceded by chronic diarrhea, worsening fatigue, and a 40 pounds weight loss over three years which were trivialized by the patient. He had neither HIV nor tuberculosis risk factors.

Physical examination revealed conjunctival pallor, diffuse abdominal pain and dehydration without fever or organomegaly. Blood glucose levels were normal, as well as the urine dipstick.

Blood tests identified metabolic acidosis (pH = 7.35, paCO _2_ = 22.9 mmHg, HCO _3_^**-**^ = 12.9 mmol/L, Base excess = -9.9, paO _2_ = 88 mmHg) with normal anion gap (about 16) and which was related to gastrointestinal loss of bicarbonate. We also noted normochromic normocytic anemia (haemoglobin = 5.7 g/dL, MCV = 81 fl, MCHC = 30 g/dL), and normal WBC (7200/μL) and platelet (174000/μL) counts. Renal and liver functions were correct (creatinine = 72 μmol/L, total bilirubin = 13.6 μmol/L, AST/ALT/ALP = 31/31/140 IU/L). The blood ionogram showed hypokalaemia (2.81 mmol/L) and hypophosphatemia (0.32 mmol/L) with normal natremia (139 mmol/L) and chloremia (110 mmol/L). Additional investigations revealed normal level of vitamin B1, negative viral serologies (Hbs, HCV and HIV) and negative toxicology work-up. Brain MRI and abdominal CT scan were normals.

The most likely cause of confusion was the dehydration after ruling out cerebral causes, toxic causes, uraemia, hepatic encephalopathy, and Gayet wernicke encephalopathy (no chronic alcoholism, no diabetes history and normal level of vitamin B1). Furthermore, we noted an improvement on the state of consciousness after hydro-electrolytic resuscitation and acid-base disorders correction. Despite the neurological improvement, the patient continued to have watery diarrhea, abdominal meteorism and weight loss. Thus, we first discussed infectious causes and the patient was treated for a possible small bowel bacterial overgrowth with an association of a third generation cephalosporin and metronidazole. The result of coproculture was negative and the parasitological stool testing wasn’t available in our laboratory. Despite antibiotherapy, he continued to have three-five loose stools daily. Autoimmune enteropathy was unlikely as no extra-intestinal manifestation were documented. Furthermore, medication-induced enteropathy was not discussed since the patient wasn’t under any treatment.

A nutritional assessment was performed and showed a malabsorption syndrome: iron deficiency (ferritin = 0.84 μg/L, serum iron = 2.44 μmol/L), hypoalbuminemia (22 g/L) and hypocalcaemia (1.8 mmol/L). We also noticed slight deficiencies in Vitamin B12: 170 pg/mL (normal: 180-914 pg/mL) and folate: 3.2 μg/L (normal > 3.5 μg/L).

**Figure 1.**
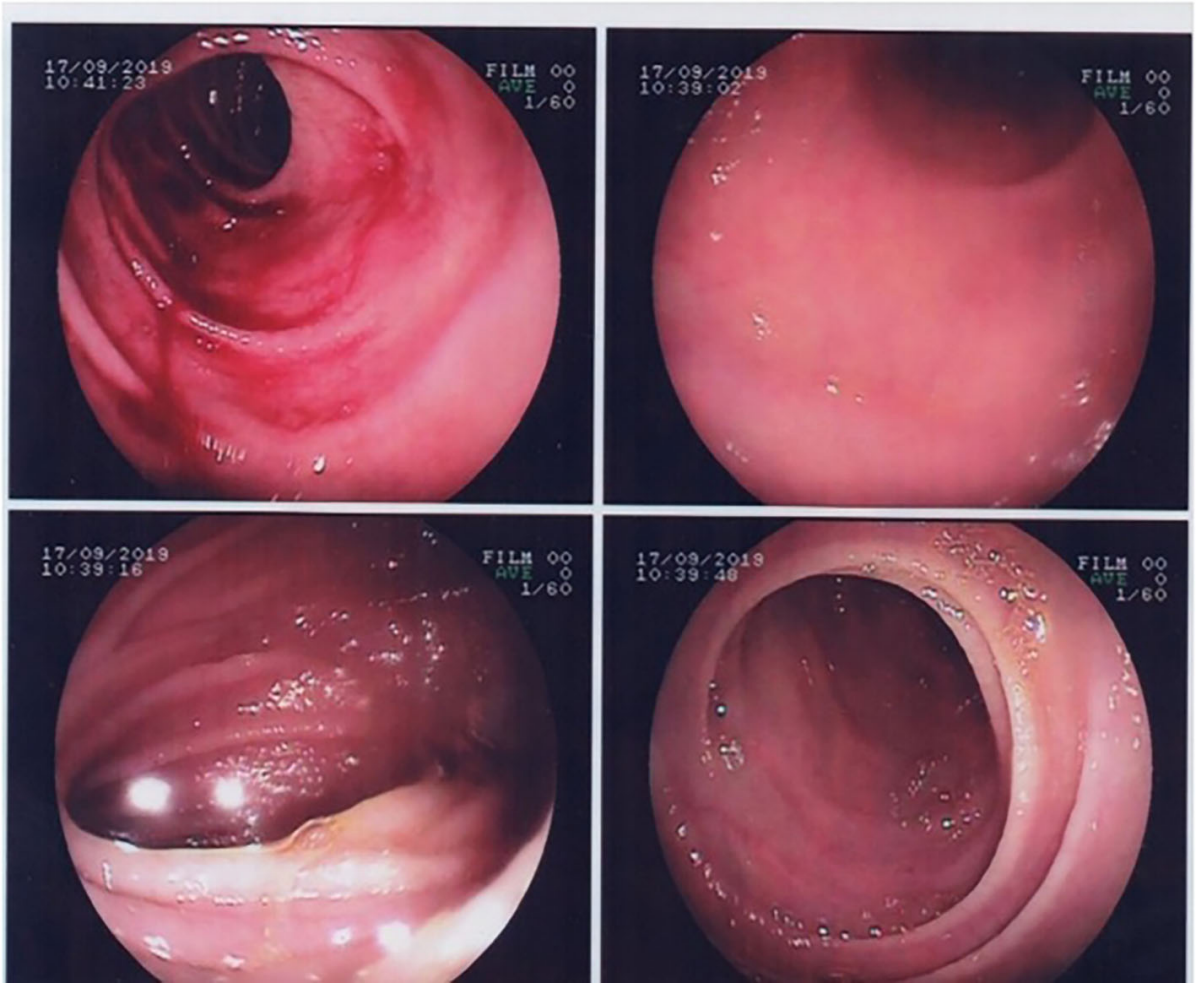
Gastrointestinal endoscopy showing duodenal villous atrophy.

Upper gastrointestinal endoscopy showed partial villous atrophy ( Figure 1). These changes were nonspecific but were suggestive of celiac disease. Thus, a gluten-free diet was prescribed.

**Figure 2.**
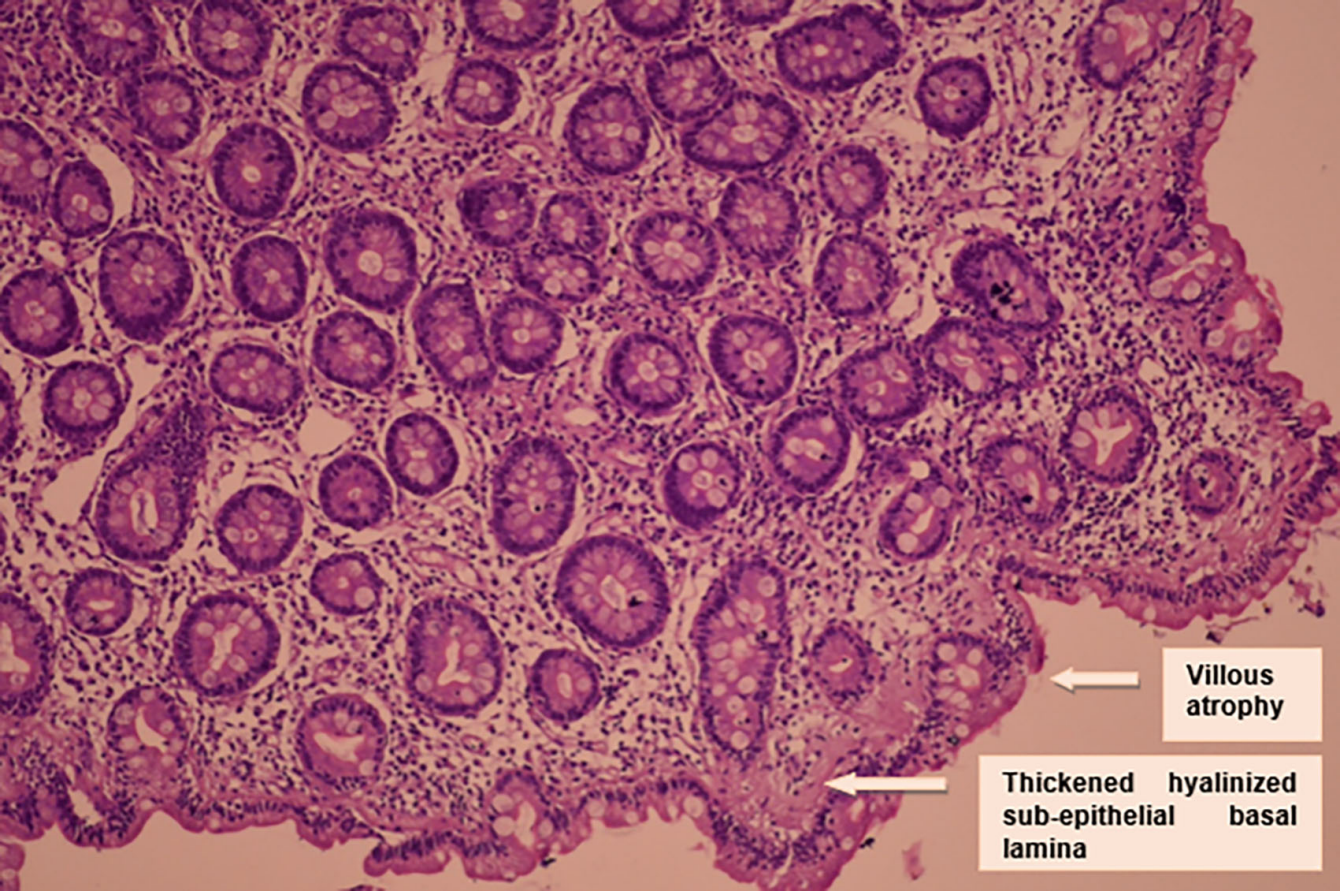
Histological findings.

Duodenal biopsy showed subtotal villous atrophy with increased intraepithelial lymphocytosis and a very thickened hyalinized sub-epithelial basal lamina: an appearance suggestive of sprue ( Figure 2).

On re-interviewing, we discovered that the patient had spent two months in India (for commercial purposes) three years prior. A few months after his return, he developed chronic intermittent watery, non-bloody diarrhea. He was also experiencing asthenia and had a weight loss but he did not consult any doctor.

Celiac serologies, including endomysial antibody, tissue transglutaminase antibody, and gliadin IgA, gliadin IgG, and total IgA levels were all negative. In light of these negative serologies and lack of response to a gluten-free diet, celiac disease was unlikely. Given the travel history, clinico-biological, endoscopic and histological findings, TS was highly considered.

A five-month antibiotic course was prescribed (tetracycline 250 mg four times daily) combined to a nutritional supplementation (iron: 150 mg and folate: 5 mg orally daily with cyanocobalamin 1000 μg daily intramuscularly for ten days, followed by 1000 μg monthly for one year). Within one month the patient reported feeling significantly better with resolution of diarrhea, increased energy and weight gain. The biochemical parameters had normalized at the three-month check-up (haemoglobin = 9.4 g/dL, and albumin = 43 g/L). The response to treatment supported the diagnosis of TS.

On the one-year follow-up visit, the patient was continuing to improve. He gained 35 pounds of weight and the diarrhea was completely gone.

## Discussion

The main strengths associated to this case report of TS were: the uncommon presentation of TS which was revealed by a confusional syndrome; and the clinico-biological, endoscopic and histological features supporting this rare and unrecognized diagnosis in non-endemic areas. Response to combined treatment confirmed the diagnosis but the lack of an endoscopic control after improvement was a limitation in our case. In fact, TS is a disease of the small intestine characterized by a malabsorption syndrome with a subtotal or partial mucosal atrophy. It occurs mainly in the tropics, particularly in most of the Greater Antilles, the northern part of South and Central America and South-East Asia. It appears to be rare in Africa, but its real frequency is unknown as small bowel biopsies are not routinely done. ^2^^,^^5^ TS affects indigenous inhabitants and expatriates, either long-term residents or short-term visitors, in these endemic areas (the case of our patient). ^5^^,^^6^ Patients can present with diarrhea soon after returning from the tropics, but rarely TS may be latent for months to years after leaving the endemic region, as was the case of our patient. ^7^

The risk factors involved in the pathogenesis of this small bowel disease are immune deficiency, poor hygiene, and bacterial, viral or parasitic gastrointestinal infections. ^4^ In fact, the most incriminated bacteria are *Klebsiella pneumoniae*, *Escherichia coli* and *Enterobacter cloacae.* ^8^^,^^9^ Enterotoxin production by some strains of enterotoxigenic *K. pneumonia* or *E. coli* can lead to abnormalities of mucosal structure and function. ^5^ Otherwise, the local action of unabsorbed bile acids might also be involved. ^4^

The diagnosis of TS is difficult. It is based on the combination of clinico-biological, histological and evolutionary criteria: ^4^^,^^8^^,^^10^

• Compatible clinical presentation: diarrhea, weight loss, asthenia,
• Evidence of a malabsorption syndrome of two unrelated substances,
• Abnormal small intestinal mucosal histology,
• Exclusion of other intestinal diseases with similar presentation,
• Improvement after treatment with tetracycline and folic acid.

Therefore, TS must be considered in patients who have lived in an endemic area, presenting with chronic diarrhea and evidence of malabsorption. ^2^ Megaloblastic anemia is common and is secondary to folate and vitamin B12 deficiencies. Associated iron deficiency may turn a macrocytic anemia into a normocytic anemia. This could explain the normochromic normocytic anemia of our patient further, since he had a history of iron deficiency anemia. Calcium, vitamin D and magnesium-impaired absorption may also occur, with resulting osteopenia. Steatorrhea is often evident if fecal fat is measured. Furthermore, abnormal D-xylose test supports proximal small intestine malabsorption. ^4^^,^^5^^,^^7^^,^^10^^,^^11^

Gastrointestinal endoscopic findings are non-specific in TS. In fact, celiac disease must be considered, especially given the endoscopic abnormalities and histological similarity with TS. ^9^^,^^12^

Biopsy from the distal portion of the duodenum reveals villous atrophy and an increased infiltration of the lamina propria by chronic inflammatory cells (plasma cells and lymphocytes). ^9^ Other causes of malabsorption (celiac disease, Crohn’s disease, bacterial overgrowth, autoimmune enteropathy, medication-induced enteropathy and lymphoma) must be ruled out. ^4^^,^^5^^,^^10^^,^^12^ Although seronegative coeliac disease is rare but possible, ^11^ our patient didn’t improve after a gluten free diet which was another argument against this diagnosis. Infectious, immune and iatrogenic causes were also unlikely in our case.

The key principles in the management of TS include rehydration, micronutrient deficiency replacement, oral folate, intramuscular vitamin B12 and antibiotics. Sulfonamides and, more recently, new quinolones, particularly ofloxacin, were tried in some previous cases reported in the literature. However, tetracycline is the antibiotic of choice, administered at a dose of 250 mg orally four times daily. ^4^^,^^5^^,^^9^^–^^11^^,^^13^ The duration of treatment has not yet been codified, as it depends on the evolution of the disease. In general, it’s a prolonged antibiotic course over three to six months. It will be stopped after control of the restitution ad integrum of the clinico-biological and histological anomalies. ^9^^,^^10^^,^^14^ In our case, tetracycline was prescribed for five months and the favourable response to treatment supported the diagnosis of TS, but a control by gastrointestinal endoscopy wasn’t carried out.

Complete resolution after an optimal management is standard in the returning travellers from an endemic area. However, for the tropics inhabitants, TS may relapse, requiring a prolonged follow-up given the risk of re-exposure to the infectious agent. ^4^^,^^7^^,^^9^^,^^14^

## Conclusions

Clinico-biological, endoscopic and histological findings overlap between TS and other malabsorption diseases, explaining diagnosis difficulties. While TS is common in tropics inhabitants, it must be considered in tropics visitors presenting with chronic diarrhea after ruling out other causes. Improvement after an optimal management combining rehydration, replacement of micronutrient, folate and vitamin B12 deficiencies as well as a prolonged antibiotic course supports the diagnosis of TS.

## Data availability

All data underlying the results are available as part of the article and no additional source data are required.

## Consent

Written informed consent for publication of the clinical details and clinical images was obtained from the patient.

## References

[1] Bartholomew C : William Hillary and sprue in the Caribbean: 230 years later. *Gut.* 1989; 30 Spec No: 17–21. 10.1136/gut.30.spec_no.17 2691344 PMC1440696

[2] Louis-Auguste J Kelly P : Tropical enteropathies. *Curr. Gastroenterol. Rep.* 2017;19(7):29. 10.1007/s11894-017-0570-0 28540669 PMC5443857

[3] Klipstein FA Baker SJ : Regarding the definition of tropical sprue. *Gastroenterology.* 1970;58:717–721. 10.1016/S0016-5085(70)80133-0 5444177

[4] Ghoshal UC Srivastava D Verma A : Tropical Sprue in 2014: the New Face of an Old Disease. *Curr. Gastroenterol. Rep.* 2014;16(6):391. 10.1007/s11894-014-0391-3 24781741 PMC7088824

[5] Klipstein FA : Tropical sprue in travelers and expatriates living abroad. *Gastroenterol.* 1981;80:590–600. 10.1016/0016-5085(81)90025-1 7450451

[6] Prendki V Grandière-Perez L Ansart S : Tropical sprue in two foreign residents, with evidence of Tropheryma whippelii in one case. *Travel Med.* 2006;13:175–177. 10.1111/j.1708-8305.2006.00038.x 16706950

[7] Ramakrishna BS Venkataraman S Mukhopadhya A : Tropical malabsorption. *Postgrad. Med. J.* 2006;82:779–787. 10.1136/pgmj.2006.048579 17148698 PMC2653921

[8] Macaigne G Boivin JF Auriault ML : Sprue tropicale: à propos de 2 cas observés dans la région parisienne. *Gastroenterol. Clin. Biol.* 2004;28:913–916. GCB-10-2004-28-10-0399-8320-101019-ART14. 10.1016/S0399-8320(04)95158-5 15523231

[9] Ranjan P Ghoshal UC Aggarwal R : Etiological spectrum of sporadic malabsorption syndrome in northern Indian adults at a tertiary hospital. *Indian J. Gastroenterol.* 2004;23:94–98. 15250566

[10] Klipstein FA Corcino JJ : Factors responsible for weight loss in tropical sprue. *Am. J. Clin. Nutr.* 1977;30:1703–1708. 10.1093/ajcn/30.10.1703 910746

[11] Westergaard H : Tropical Sprue. *Curr. Treat. Options Gastroenterol.* 2004;7(1):7–11. 10.1007/s11938-004-0020-6 14723833

[12] Green PHR Paski S Ko CW : AGA Clinical practice update on management of refractory celiac disease: Expert Review. *Gastroenterology.* 2022;163(5):1461–1469. 10.1053/j.gastro.2022.07.086 36137844

[13] Walker MM : What is tropical sprue? *J. Gastroenterol. Hepatol.* 2003;18:887–890. 10.1046/j.1440-1746.2003.03127.x 12859716

[14] Rickles FR Klipstein FA Tomasini J : Long-term follow-up of antibiotic-treated tropical sprue. *Ann. Intern. Med.* 1972;76:203–210. 10.7326/0003-4819-76-2-203 5009590

