# Supplementary material for: Case Report: Tropical sprue, diagnostic challenges of an old but unrecognized disease
Source: F1000Res. 2024 Aug 20;11:1206. Originally published 2022 Oct 24. [Version 3] doi: 10.12688/f1000research.125305.3 (PMC11359971; doi:10.12688/f1000research.125305.3)
Supplement: Peer review history

# Reviewer report: Reviewer response for version 3

**DOI:** 10.5256/f1000research.170343.r315485

**Published:** 2024-08-28

**Authors:** Laisa Socorro Briongos-Figuero

**Affiliations:** 1 Rio Hortega University Hospital, Valladolid, Spain

I think changes made a add great quality to this case and are appropriate.

Are enough details provided of any physical examination and diagnostic tests, treatment given and outcomes?

Partly

Is the case presented with sufficient detail to be useful for other practitioners?

Yes

Is sufficient discussion included of the importance of the findings and their relevance to future understanding of disease processes, diagnosis or treatment?

Yes

Is the background of the case’s history and progression described in sufficient detail?

Yes

Reviewer Expertise:

Infectious diseases; Internal Medicine

I confirm that I have read this submission and believe that I have an appropriate level of expertise to confirm that it is of an acceptable scientific standard.

# Reviewer report: Reviewer response for version 3

**DOI:** 10.5256/f1000research.170343.r315484

**Published:** 2024-08-24

**Authors:** Wolfgang Kreisel

**Affiliations:** 1 Department of Medicine II, Gastroenterology, Hepatology, Endocrinology and Infectious Diseases, Faculty of Medicine, University of Freiburg, Freiburg, Baden-Württemberg, Germany

The case report can be in the indexed in the revised form.

Are enough details provided of any physical examination and diagnostic tests, treatment given and outcomes?

Partly

Is the case presented with sufficient detail to be useful for other practitioners?

Yes

Is sufficient discussion included of the importance of the findings and their relevance to future understanding of disease processes, diagnosis or treatment?

Yes

Is the background of the case’s history and progression described in sufficient detail?

Yes

Reviewer Expertise:

NA

I confirm that I have read this submission and believe that I have an appropriate level of expertise to confirm that it is of an acceptable scientific standard.

# Reviewer report: Reviewer response for version 2

**DOI:** 10.5256/f1000research.155449.r292008

**Published:** 2024-06-24

**Authors:** Laisa Socorro Briongos-Figuero

**Affiliations:** 1 Rio Hortega University Hospital, Valladolid, Spain

The study of the spectrum of malabsorption could be arduous in chronic diarrhea. I have carefully revised the manuscript and I think it's a worthy, well-structured and interesting case report but I miss some information:

 - TS is a rare cause of malabsorption syndrome characterized by  megaloblastic anemia due to vitamin B12 deficiency. The patient had a mild B12 deficiency and a severe iron deficiency. Could you explain your thoughts about this fact?

 - Neurological disorders could be a symptom of other conditions like Whipple's disease, tuberculosis or common variable immunodeficiency enteropathy. Did a polymerase chain reaction test for Tropheryma whipplei and Mycobacterium tuberculosis underwent a on duodenal tissue or stool?

 - provided imaging evidence that the mucosal lesions had resolved at follow-up would be helpful for comparison

 The manuscript may be approved with reservations. Some changes are needed.

Are enough details provided of any physical examination and diagnostic tests, treatment given and outcomes?

Partly

Is the case presented with sufficient detail to be useful for other practitioners?

Yes

Is sufficient discussion included of the importance of the findings and their relevance to future understanding of disease processes, diagnosis or treatment?

Yes

Is the background of the case’s history and progression described in sufficient detail?

Yes

Reviewer Expertise:

Infectious diseases; Internal Medicine

I confirm that I have read this submission and believe that I have an appropriate level of expertise to confirm that it is of an acceptable scientific standard, however I have significant reservations, as outlined above.

# Response

**Published:** 2024-08-12

**Authors:** Jihene Guissouma

**Affiliations:** medical intensive care department of Habib Bougatfa teaching hospital Bizerte Tunisia, Tunisia

**Dear reviewer:**

 Thanks for the time you have devoted to review this paper and for giving us the opportunity to benefit from your expertise. Here are the answers to your comments :

 - To explain the type of anemia in our case: you are absolutely right, megaloblastic anemia is the most common form of macrocytic anemia caused by vitamin B12 and/or folate deficiency. However, in some cases reported in the literature, blood tests may reveal normocytic normochromic anemia, particularly in the early stages of macrocytic anemia or in cases of combined microcytic and macrocytic anemia. This may further explain the normochromic normocytic anemia of our patient, since he had a long history of iron deficiency responsible of a hypochromic microcytic anemia to which was added the vitamin B12 and folate deficiency.

 - To discuss other causes of neurological disorders and malabsorption conditions, we believe that the confusion was explained by metabolic disorders as the state of consciousness improved after hydroelectrolytic resuscitation and correction of acid-base disorders, even more so as the brain MRI was normal. Whipple's disease was unlikely as the patient didn’t have initially fever or arthralgia. The investigations carried out did not reveal any involvement of other organs (heart, lung, brain or serous cavities). Besides, the Histological findings were not consistent with this disease since no macrophage or bacillus were visualized in the biopsy. In addition, the patient had no history of tuberculosis or contagion and the abdominal CT scan was normal. Unfortunately, PCR test for Tropheryma whipplei and Mycobacterium tuberculosis were not available in our hospital.

 Beyond the digestive symptoms, we had no arguments in favor of common variable immunodeficiency enteropathy. The patient had no history of recurrent infections, the gamma globulin level was normal and the mucosal biopsy was not suggestive. Furthermore, improvement without immunoglobulin replacement therapy was another argument against this diagnosis.

 - We couldn’t provide imaging evidence that the mucosal lesions had resolved at follow-up. In fact, we intended to check the gastrointestinal endoscopy at follow-up, but as the patient had progressed well, he refused to undergo it.

 All these points were checked in the new version.

 We hope that we have replied to all your queries and we are available if any other points remain unclear.

**Best regards**

# Reviewer report: Reviewer response for version 2

**DOI:** 10.5256/f1000research.155449.r206204

**Published:** 2023-09-26

**Authors:** Wolfgang Kreisel

**Affiliations:** 1 Department of Medicine II, Gastroenterology, Hepatology, Endocrinology and Infectious Diseases, Faculty of Medicine, University of Freiburg, Freiburg, Baden-Württemberg, Germany

I confirm that the present version may be indexed.

Are enough details provided of any physical examination and diagnostic tests, treatment given and outcomes?

Partly

Is the case presented with sufficient detail to be useful for other practitioners?

Yes

Is sufficient discussion included of the importance of the findings and their relevance to future understanding of disease processes, diagnosis or treatment?

Yes

Is the background of the case’s history and progression described in sufficient detail?

Yes

Reviewer Expertise:

NA

I confirm that I have read this submission and believe that I have an appropriate level of expertise to confirm that it is of an acceptable scientific standard.

# Reviewer report: Reviewer response for version 1

**DOI:** 10.5256/f1000research.137598.r178343

**Published:** 2023-06-20

**Authors:** Wolfgang Kreisel

**Affiliations:** 1 Department of Medicine II, Gastroenterology, Hepatology, Endocrinology and Infectious Diseases, Faculty of Medicine, University of Freiburg, Freiburg, Baden-Württemberg, Germany

Tropical sprue is a frequent cause of malassimilation syndrome in tropical countries accounting for up to 30% of affected persons. However, it is rare in Europe, North America, or Countries lining the Mediterranean Sea. The authors describe the case of a 58-years-old male patient who presented with confusion, dehydration, and watery diarrhea in a University Hospital in Tunisia. Clinical presentation, endoscopy of the duodenum, and thorough anamnestic evaluation (the patient spent 2 months in India), and response to antibiotic therapy led to the correct diagnosis of tropical sprue. The paper is very interesting, and it is well written. However, I recommend stylistic editing for English language and grammar.

 Several items should be corrected or changed:

- The patients had only a mild metabolic acidosis, not a severe acidosis. The blood pH of 7.35 is at the lower limit of normal. Please indicate base excess and exact value for anion gap. Metabolic acidosis with normal anion gap proves bicarbonate loss, as the authors indicate. The confusion is rather the consequence of dehydration that the consequence of the mild (!) acidosis. The hypokalemia of 2.81 mMol/L in acidosis is a dangerous situation.
- Further blood tests clearly indicated malassimilation syndrome, as was further demonstrated by the evidence of villous atrophy. The authors use the term “hyalonalized”. Is this a generally accepted term? Should it be “hyaluronalized”? Amorphic material beneath the basal membrane resembling hyaluronic acid?
- I suggest to better discuss the differential diagnosis of villous atrophy (celiac disease, refractory celiac disease, autoimmune enteropathy, post-infectious villous atrophy etc.). Was giardiasis excluded, or other intestinal pathogens?
- Therapy and clinical course are correct described.
- As for refs., I recommend to add few newer reviews, e.g. Louis-Auguste & Kelly (2017) ^1^;. Green *et al.*, (2022) ^2^.

The manuscript may be approved with reservations.

Are enough details provided of any physical examination and diagnostic tests, treatment given and outcomes?

Partly

Is the case presented with sufficient detail to be useful for other practitioners?

Yes

Is sufficient discussion included of the importance of the findings and their relevance to future understanding of disease processes, diagnosis or treatment?

Yes

Is the background of the case’s history and progression described in sufficient detail?

Yes

Reviewer Expertise:

Gastroenterology. Infectious Diseases. Celiac Disease

I confirm that I have read this submission and believe that I have an appropriate level of expertise to confirm that it is of an acceptable scientific standard, however I have significant reservations, as outlined above.

## References

[1] : Tropical Enteropathies. *Curr Gastroenterol Rep* .2017;19(7) : 10.1007/s11894-017-0570-0 29 10.1007/s11894-017-0570-0 28540669 PMC5443857

[2] : AGA Clinical Practice Update on Management of Refractory Celiac Disease: Expert Review. *Gastroenterology* .2022;163(5) : 10.1053/j.gastro.2022.07.086 1461-1469 10.1053/j.gastro.2022.07.086 36137844

# Response

**Published:** 2023-08-31

**Authors:** Jihene Guissouma

**Affiliations:** medical intensive care department of Habib Bougatfa teaching hospital Bizerte Tunisia, Tunisia

Dear Reviewer

 Thank you for accepting to review this article.

 We agree that the metabolic acidosis wasn’t severe in our case. The base excess and the anion gap values were -9,9 and 16 respectively.

 We corrected the term hyalonalized with hyalinized.

 We have better discussed the differential diagnosis. In fact, bacterial overgrowth was unlikely as the coprocultrure were negative and the patient didn’t improve after antibiotherapy.

 Unfortunately, parasitological stool testing wasn’t available in our laboratory. Autoimmune enteropathy was unlikely as no extra-intestinal manifestation were documented. Furthermore, medication-induced enteropathy was not discussed since the patient wasn’t under any treatment. Although seronegative celiac disease is rare but possible our patient didn’t improve after gluten free diet which was another argument against this diagnosis.

 We added the new reviews recommended.
